# Encrusted Cystitis in a Child Without Predisposing Factors: A Case Report and Literature Review

**DOI:** 10.7759/cureus.84576

**Published:** 2025-05-21

**Authors:** Shohei Yoshimura, Kengo Hattori, Emi Tsuji, Jiro Tsugawa

**Affiliations:** 1 Department of Pediatric Surgery, Takatsuki General Hospital, Takatsuki, JPN; 2 Department of Pediatric Surgery, Hyogo Prefectural Kobe Children's Hospital, Kobe, JPN

**Keywords:** bladder and bowel dysfunction, encrusted cystitis, pediatric constipation, recurrent rectal prolapse, urinary tract infection

## Abstract

A six-year-old girl was admitted to our hospital with rectal prolapse, urinary frequency, urinary stone drainage, and recurrent febrile urinary tract infections. A urinary culture identified *Proteus mirabilis*, and an ultrasonography demonstrated mucosal calcification of the bladder wall, leading to the diagnosis of encrusted cystitis. Antibiotic administration and transurethral resection of bladder calcification were performed, and her symptoms gradually disappeared. Encrusted cystitis is extremely rare in childhood and is frequently observed in patients with post-renal transplantation, urological interventions, and immunosuppressive status. She did not have these predisposing factors; thus, bladder and bowel dysfunction may be a potential risk factor for encrusted cystitis.

## Introduction

Encrusted cystitis (EC) is a rare chronic inflammatory condition of the bladder, clinically characterized by the formation of struvite calcifications of the bladder mucosa [1]. These calcifications are caused by the action of urea-splitting bacteria, including species such as *Corynebacterium*, *Proteus*, *Klebsiella*, and *Pseudomonas* [2]. These bacteria produce ammonia through the urea hydrolysis, which disrupts the glycosaminoglycan layer of the bladder, resulting in the formation of struvite stones and calcification of the bladder wall [3].

A history of renal transplantation, previous urological intervention, bladder catheterization, or immunosuppressed status has been identified as a potential risk factor for the development of EC in both adult and pediatric populations [1,4]. Consequently, EC without these predisposing factors is exceedingly rare in the literature, with only one adult case having been previously reported [3]. In this case report, we present a pediatric case of EC that lacks any reported cofactors.

## Case presentation

**Figure 1:**
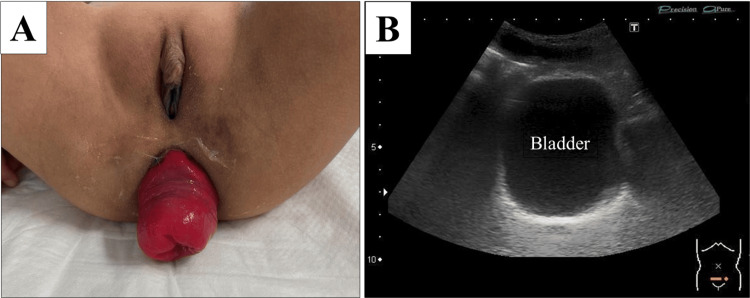
Physical and ultrasonographic findings. (A) Rectal prolapse in the outpatient clinic. (B) Ultrasound of the bladder showing a 5-mm-thickened calcification of the entire bladder wall.

A six-year-old girl with an autism spectrum disorder presented to our outpatient clinic with a two-month history of recurrent rectal prolapse (Figure 1A). Moreover, the patient exhibited symptoms of urinary frequency, suprapubic pain, an unpleasant odor in the urine, and urinary stones. She had no history of immunodeficiency or immunosuppressive treatment and no medication for autism spectrum disorder. Magnesium oxide had been administered for four months to treat constipation. The urinalysis yielded a pH of 9.0, hematuria, and proteinuria in the outpatient clinic. Additionally, the calculus analysis indicated the presence of 83% ammonium magnesium phosphate calculus, also known as struvite stones. Despite the replacement of magnesium oxide with polyethylene glycol, her symptoms persisted for a few months, accompanied by recurrent febrile urinary tract infections (UTIs). A urinary culture obtained during a febrile UTI detected 1 × 10^5^ colony-forming units/mL *Proteus mirabilis*, which was identified as the causative bacterium. An abdominal ultrasound revealed the presence of calculi deposition on the bladder wall (Figure 1B), which led to the diagnosis of EC.

**Figure 2:**
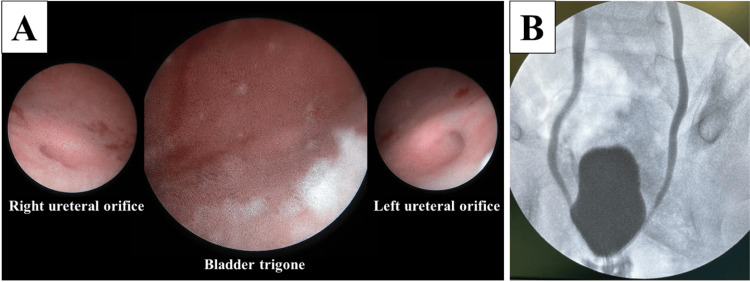
Intraoperative findings. (A) Cystoscopy showing calcium deposits centered on the bladder trigone and sclerosis of the bilateral ureteral orifices. (B) Intraoperative cystography showing secondary bilateral vesicoureteral reflux.

A cystoscopy under general anesthesia demonstrated calcium deposits concentrated around the bladder trigone and sclerosis of the bilateral ureteral orifices (Figure 2A). Intraoperative cystography confirmed secondary bilateral vesicoureteral reflux (Figure 2B). In addition to intravenous antibiotics administration for *Proteus mirabilis* for four weeks, transurethral resection of the bladder wall calcification was performed. Her symptoms cleared gradually, and the follow-up urinalysis revealed a normal pH level and no evidence of hematuria or proteinuria. The calcium deposit on the bladder wall observed on the ultrasonography was no longer present eight months later. She had no recurrence of rectal prolapse and UTI for more than one year.

## Discussion

EC is a rare chronic UTI associated with bladder wall calcification, first described in 1914 by Francois [5]. It is most prevalent in elderly patients or those with predisposing risk factors, including a history of renal transplantation, urological procedures, or immunosuppressive medications [1,4]. The diagnosis of EC in childhood is exceedingly rare, with only seven pediatric cases (median age at diagnosis: nine years, male-to-female ratio 4:3) having been previously reported in the English literature, including our case (Table 1) [6-9]. Among the seven patients, three (43%) had undergone prior urological interventions, two (29%) had a history of renal transplantation, and one (14%) was on long-term corticosteroid therapy. To the best of our knowledge, this is the only reported pediatric case of EC reported without predisposing factors.

**Table 1:** Previously reported cases of encrusted cystitis diagnosed in children under 16 years of age. *C. urealyticum*, *Corynebacterium urealyticum*; F, female; M, male; *P. mirabilis*, *Proteus mirabilis*

Case	Age	Gender	Background	Microorganism	Antibiotics	Resection	Acidification
1 [6]	8	M	Post-renal transplantation	C. urealyticum	Yes	Open	Yes
2 [7]	9	M	Ascending pyelography for ectopic kidney	C. urealyticum	Yes	No	No
3 [8]	9	M	Bladder neck operation for bladder exstrophy	C. urealyticum	Yes	Transurethral	No
4 [8]	12	F	Bladder biopsy for Hinman syndrome	C. urealyticum	Yes	Transurethral	No
5 [8]	13	M	Post-renal transplantation	C. urealyticum	Yes	No	Yes
6 [9]	15	F	High-dose steroid use for hereditary angioedema	C. urealyticum	Yes	Transurethral	No
Present case	6	F	Rectal prolapse, no urological intervention	P. mirabilis	Yes	Transurethral	No

In the present case, rectal prolapse resulting from uncontrolled constipation was considered the primary cause of EC, which indicated a correlation with the pathophysiology of bladder and bowel dysfunction (BBD). BBD is typified by lower urinary symptoms such as dysuria and urinary frequency, in conjunction with bowel complaints as represented by constipation, and is frequently seen in children diagnosed with behavioral and neuropsychiatric disorders, such as autism spectrum disorder [10]. As a mechanism of BBD, rectal distension due to chronic constipation has been demonstrated to exert mechanical and neurological effects on the capacity, contractility, and sensation of the bladder [11]; therefore, in our case, chronic constipation with rectal prolapse affected bladder dysfunction, resulting in the growth of urinary tract bacteria. *Corynebacterium urealyticum* has been recognized as the most prevalent causative microorganism for EC, and *Proteus mirabilis*, a urea-splitting bacterium, has also been identified as a causative microorganism for EC. Ammonia produced from urea hydrolysis has been observed to alkalinize and disrupt the glycosaminoglycan layer of the bladder mucosa, thereby facilitating bacterial adhesion [3]. Ultimately, this process leads to the formation of struvite stones and bladder wall calcification, which in turn contribute to the development of EC.

The presence of bowel dysfunction, including constipation and fecal incontinence, has not been described among the seven patients in this study. However, Hinman syndrome is a known disease that can lead to BBD [10]. Therefore, although case 4 had a traumatic event of bladder biopsy one year before the development of EC [8], the patient may have developed EC through a similar mechanism as our case. In the future, an increase in EC cases may reveal an association between EC and BBD. Furthermore, routine evaluation of bowel habits may contribute to the diagnosis, particularly in pediatric patients with recurrent UTIs and lower urinary tract symptoms.

As treatments for EC, the most common approaches are the administration of appropriate antibiotics against microorganisms, the elimination of calcareous incrustation, and urine acidification [2]. Our review of the literature revealed that all patients required antibiotics for the causative bacterium, and 5/7 (71%) underwent transurethral or open resection of the bladder wall calcification. Urine acidification was indicated for only 2/7 (29%) patients, both of whom had both EC and encrusted pyelitis following post-renal transplantation. In one of these patients (case 5), nephrostomy and graft removal were also necessary. However, the prognosis was favorable, with a curable condition in all patients. In our case, optimizing bowel function in addition to antibiotic administration and transurethral resection of the bladder wall calcification was crucial to achieving a cure of EC.

## Conclusions

We showed a pediatric EC case without predisposing cofactors such as renal transplantation, urinary interventions, and immunosuppressants. BBD is a possible associated factor for EC in children, and an accumulation of EC cases may reveal an association between EC and BBD in the near future.
